# Investigation of Infection of *Enterocytozoon bieneusi* and *Giardia duodenalis* in Beef Cattle in Yunnan, China

**DOI:** 10.3390/vetsci12060552

**Published:** 2025-06-05

**Authors:** Fan Yang, Wenjie Cheng, Jianfa Yang, Junjun He, Liujia Li, Fengcai Zou, Fanfan Shu

**Affiliations:** 1 The Yunnan Key Laboratory of Veterinary Etiological Biology, Yunnan Agricultural University, Kunming 650201, China; yangffan1026@sina.com (F.Y.); cwj210365@163.com (W.C.); jsc315@163.com (J.Y.); hejunjun617@163.com (J.H.); 2 College of Veterinary Medicine, Yunnan Agricultural University, Kunming 650201, China; 3 College of Agriculture and Biological Science, Dali University, Dali 671003, China; liliujia2007@163.com

**Keywords:** *Enterocytozoon bieneusi*, *Giardia duodenalis*, beef cattle, genotypes, China

## Abstract

*Enterocytozoon bieneusi* and *Giardia duodenalis* are major zoonotic pathogens that often cause diarrhea in immunocompromised humans or animals. Beef cattle are important reservoirs for these two pathogens. Yunnan Province is a major region for beef cattle farming, and its suitable climatic conditions facilitate the transmission of the pathogens. However, research on the prevalence and distribution of *E. bieneusi* and *G. duodenalis* in beef cattle in Yunnan remains understudied. This study collected 529 fecal samples from seven beef cattle breeds in four regions in Yunnan Province for an epidemiological survey. Nested PCR combined with sequencing was used to detect *E. bieneusi* and *G. duodenalis*, and the sequencing results were analyzed to determine genotypes or assemblage types. Our results demonstrate that the prevalence rates of *E. bieneusi* and *G. duodenalis* were 3.0% (16/529) and 3.6% (19/529), respectively. The study identified four genotypes of *E. bieneusi*, including I (*n* = 8), J (*n* = 4), BEB8 (*n* = 3), and BEB4 (*n* = 1). Both assemblages E (*n* = 18) and A (*n* = 1) were identified among *G. duodenalis*-positive animals. Phylogenetic analysis revealed that the *E. bieneusi* genotypes detected in this study belong to Group 2. In conclusion, these findings indicate that although the overall prevalence is relatively low compared to other regions, the presence of zoonotic Group 2 genotypes and assemblage A highlights the potential risk of cross-species transmission. Moreover, the results provide foundational data to support the development of region-specific surveillance and control strategies for bovine giardiasis and microsporidiosis, and emphasize the importance of the One Health approach in managing parasitic infections in livestock populations.

## Simple Summary

*Enterocytozoon bieneusi* and *Giardia duodenalis* are significant zoonotic protists that infect humans and animals worldwide. Cattle are common hosts for these pathogens. Understanding the genetic diversity of these pathogens in beef cattle is crucial for disease prevention and control. This study has investigated the prevalence and genotypes of *E. bieneusi* and assemblages of *G. duodenalis* in seven beef cattle populations across four regions of Yunnan Province, China. The results demonstrate that the prevalence rates of *E. bieneusi* and *G. duodenalis* were 3.0% (16/529) and 3.6% (19/529), respectively. These two pathogens infection rates showed significant differences among animals of different age groups. Four *E. bieneusi* genotypes and two *G. duodenalis* assemblages were identified. This study reveals the prevalence of *E. bieneusi* and *G. duodenalis* in beef cattle in Yunnan Province for the first time. The findings provide essential baseline data for developing effective prevention and control strategies against these parasitic infections in the region’s cattle industry.

## 1. Introduction

*Enterocytozoon bieneusi* and *Giardia duodenalis* are globally distributed zoonotic protozoa that can infect a variety of vertebrates, including humans, livestock, and wildlife [1,2]. These pathogens are primarily transmitted through the fecal–oral route, with contaminated food and water serving as major vectors of infection [3,4]. Infection with these pathogens is typically asymptomatic in immunocompetent hosts, while immunocompromised individuals (e.g., HIV-positive patients, organ transplant recipients, children, and the elderly) may experience symptoms ranging from self-limiting diarrhea to severe wasting syndrome [5,6,7,8,9]. Recent studies have highlighted growing concerns about the role of cattle as reservoirs for these pathogens, given their close contact with humans and the environment they share [10].

The detecting of *E. bieneusi* accurately under the microscope is challenging, making the PCR method a common and effective approach for its identification [10]. The internal transcribed spacer (ITS) region of the rRNA gene servers as the primary genetic marker for *E. bieneusi* genotyping [11]. Based on ITS sequence variation, over 500 genotypes have been identified and classified into 15 phylogenetic groups [12,13]. Group 1 and 2 genotypes demonstrate public health risks, as they include genotypes with broad host range and zoonotic potential, which is the ability to transmit between animals and humans, whereas Groups 3–15 exhibit strict host specificity [3,14]. Current studies have identified over 40 *E. bieneusi* genotypes in cattle, predominantly from Group 2 [15,16]. Notably, 15 of these genotypes (8 from Group 1 and 7 from Group 2) have also been detected in human infections [13,15,17], indicating cattle may serve as important reservoirs for zoonotic transmission.

Similarly, *G. duodenalis* exhibits host-adapted genetic diversity, with eight assemblages (A–H) identified. Zoonotic assemblages A and B infect humans and animals, while assemblages C–H are host-specific. In cattle, assemblage E predominates as the most prevalent *G. duodenalis* genotype globally, while zoonotic assemblages A and B have been reported in some epidemiological investigations [18]. This highlights the need for surveillance in cattle populations, particularly in high-density farming regions.

Yunnan Province, located in southwestern China, is one of the country’s most important regions for beef cattle breeding. As China’s largest beef cattle production province, it maintained nearly 9 million heads of cattle in 2023, and is home to at least 15 different breeds of cattle [19]. The warm climate and intensive farming practices in Yunnan Province may facilitate the spread of pathogens. In previous studies, dairy cattle and dairy buffalo in Yunnan Province were found to be infected with *E. bieneusi*, with five genotypes identified in a small-scale survey of animals [20]. Furthermore, *G. duodenalis* assemblages A and E have been reported in both dairy cattle and Yunling cattle in Yunnan Province [21,22]. However, the prevalence of these pathogens in beef cattle in Yunnan remains understudied. The “One Health” framework highlights the need for integrated strategies to reduce disease risks at the human–animal–environment interface. A better understanding of *E. bieneusi* and *G. duodenalis* in beef cattle could inform more effective control measures. Therefore, this study aimed to determine the occurrence and their genotypes of *E. bieneusi* and *G. duodenalis* in seven cattle breeds in four regions of Yunnan, and to assess potential transmission risks to humans.

## 2. Materials and Methods

### 2.1. Sample Collection and Sampling Area

**Figure 1:**
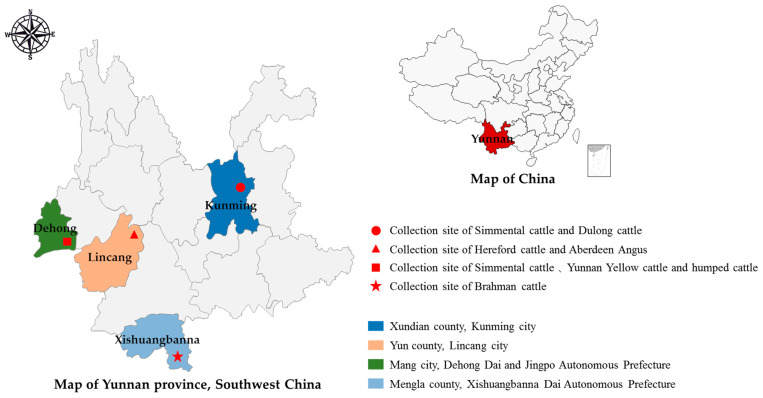
Map of beef cattle sampling sites in Yunnan Province, China.

This study collected fecal samples from seven beef cattle breeds (Simmental, Brahman, Aberdeen Angus, Yunnan Yellow, Dulong, Hereford, and Humped) in four different regions in Yunnan Province (Figure 1). A total of 160 samples were obtained from Kunming, 162 from Dehong, 106 from Xishuangbanna, and 101 from Lincang (Table 1). The sampling in Baoshan, Dehong, and Xishuangbanna took place in June in 2022, while sampling in Lincang was conducted in September, and in Kunming in October 2024. All cattle were kept in confinement and fed self-produced silage and commercial concentrate feed. All samples were freshly collected and picked up with disposable polyethylene gloves; the gloves were then turned inside out and placed into appropriately sized transparent resealable bags, while simultaneously recording the sample information and collection time. Each sample was individually packaged to avoid cross-contamination between samples. Sample information was recorded during the sampling process, and the packaged samples were then transported to the laboratory, stored at −20 °C, and tested within 48 h.

### 2.2. DNA Extraction and PCR

Prior to genomic DNA extraction, approximately 300 mg of fecal samples preserved in potassium dichromate were washed three times with distilled water through centrifugation (2000× *g* for 10 min each time). The resulting pellet was processed using the FastDNA Spin Kit for Soil (MP Biomedicals, Solon, OH, USA), following the manufacturer’s protocol [21]. The purified DNA was stored at −20 °C until subsequent PCR analysis.

### 2.3. PCR Amplification

The detection of *E. bieneusi* was performed using a nested PCR assay targeting the ITS gene, as described in reference [23] (Table 2), with an expected amplicon size of approximately 392 bp. Additionally, all fecal DNA samples were subjected to PCR amplification targeting the *gdh* locus of *Giardia* spp. following the protocol outlined in reference [24] (Table 2), yielding a product of approximately 530 bp. DNA preparations of assemblage G from mice and genotype PtEb IX from dogs were used as positive controls in the PCR analysis for *G. duodenalis* and *E. bieneusi*, respectively, whereas ultrapure water was used as the negative control. After amplification, the PCR products were analyzed by agarose gel electrophoresis and visualized using a gel imaging system.

### 2.4. Sequence Analysis

All secondary PCR products testing positive were subjected to bidirectional sequencing at Sangon Biotech (Shanghai, China) using an ABI 3730xl Genetic Analyzer (Applied Biosystems, Thermo Fisher Scientific, Foster City, CA, USA). Sequence data processing involved the following: (1) raw trace file assembly using ChromasPro 2.0 (Technelysium Pty Ltd., Queensland, Australia), (2) manual editing and quality control in BioEdit 7.3 (Ibis Therapeutics, Carlsbad, CA, USA), and (3) multiple sequence alignment against GenBank reference sequences via Clustal Omega 1.2.4 with default parameters. Genotype classification followed international standardization guidelines [24]. Phylogenetic reconstruction was performed in MEGA-11 using the maximum likelihood algorithm, with node support evaluated by 1000 bootstrap replicates.

### 2.5. Statistical Analysis

The occurrence frequencies of *E. bieneusi* and *G.duodenalis* in different regions, breeds, ages, and genders were analyzed using chi-square tests in SPSS 20.0 (IBM SPSS, Chicago, IL, USA) and SAS 9.1 (SAS Institute Inc., Cary, NC, USA), with statistical significance set at *p* < 0.05. Odds ratios (ORs) and 95% confidence intervals (CIs) were computed to evaluate potential risk factors.

## 3. Results

### 3.1. Prevalence and Genotypes of E. bieneusi

Of the 529 fecal samples collected from beef cattle, 3.0% (95% CI: 1.56–4.49%; 16/529) were tested positive for *E. bieneusi* in the PCR analysis of the ITS, with infection rates ranging from 0 to 4.4% among the four locations (Table 3). The highest infection rate at Kunming was 4.4% (95% CI: 1.17–7.58%; 7/160), followed by Dehong 3.7% (95% CI: 0.76–6.64%; 6/162) and Lincang 3.0% (95% CI: −0.40–6.34%; 3/101). No *E. bieneusi* was detected in samples from Xishuangbanna. Of the different breeds, Simmental cattle showed the highest detection rate of 5.6% (95% CI: 2.48–8.63%; 12/216), followed by Aberdeen Angus cattle 3.8% (95% CI: −0.50–8.00%; 3/80) and Humped cattle 2.8% (95% CI: −2.86–8.42%; 1/36), while no infection was found in four other breeds. By age, the infection rate in pre-weaned calves (20.7% (95% CI: 5.01–36.37%; 6/29)) was higher than that in growing cattle (6.8% (95% CI: 0.90–12.61%; 5/74)), post-weaned calves (3.2% (95% CI: −1.30–7.75%; 2/62)), and adult cattle (0.8% (95% CI: −0.11–1.76%; 3/364)). By sex, female cattle had higher infection rates of 4.0% (95% CI: 1.57–6.50%; 10/248) than males at 2.1% (95% CI: 0.43–3.84%; 6/281). Statistical analysis revealed significant differences only among age groups (*p* < 0.001), while no significant differences were observed for the other three factors (region, breed, or sex).

**Figure 2:**
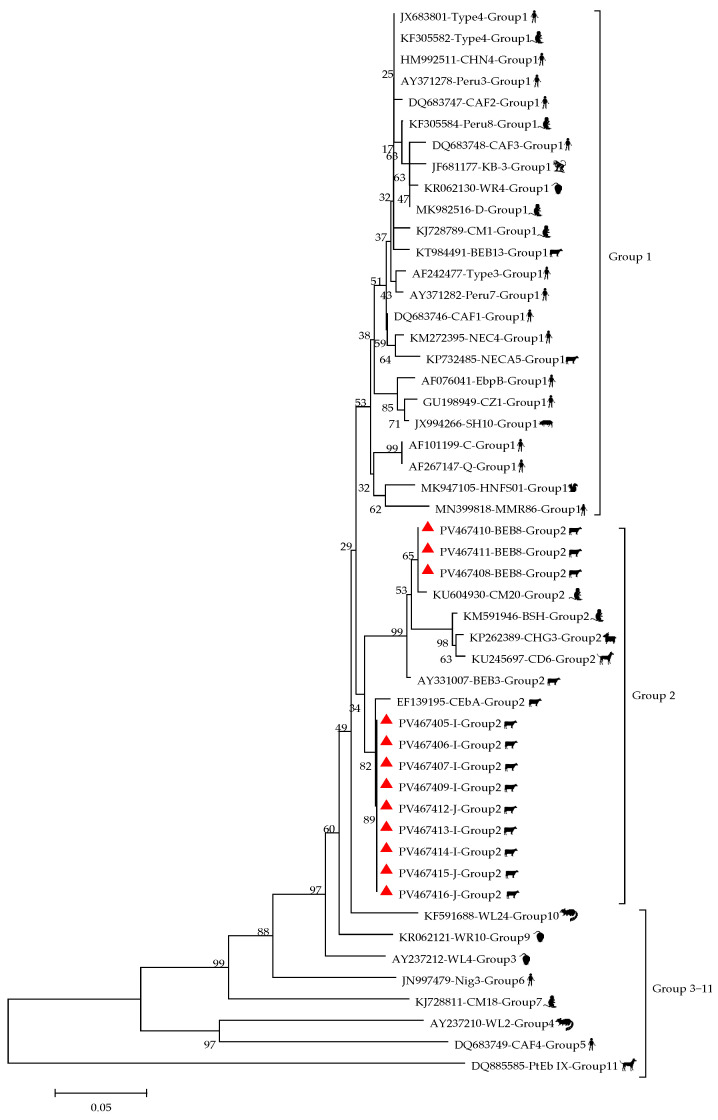
Phylogenetic tree of the *E. bieneusi* based on the ITS gene sequence. The red symbols are the samples in this study.

This study identified four genotypes of *E. bieneusi*, namely, I, J, BEB8, and BEB4. Among them, genotype I was the most frequently detected (50.0%, 8/16), followed by J (25.0%, 4/16), BEB8 (18.8%, 3/16), and BEB4 (6.3%, 1/16). Genotype I was found in Kunming, Dehong, and Lincang; genotype J was detected in Dehong and Lincang; BEB8 and BEB4 were distributed in Kunming and Dehong, respectively. By breed, genotypes I and J were primarily found in Simmental and Aberdeen Angus cattle, while all three BEB8 cases were exclusively detected in Simmental cattle. The only BEB4 case was identified in Humped cattle. By age category, genotype I was detected in all age categories except weaned calves. Genotype J was found in young cattle and weaned calves, BEB8 was present in pre- and post-weaning calves, and the single case of BEB4 was only detected in adult cattle. In terms of sex, genotypes I and J were detected in both females and males, whereas BEB8 and BEB4 were found only in females and males, respectively. Phylogenetic analysis revealed that all the genotypes obtained in this study belong to Group 2 (Figure 2). The representative sequences have been submitted to GenBank and assigned GenBank accession numbers of PV467405 to PV467416.

### 3.2. Prevalence and Genotypes of G. duodenalis

**Figure 3:**
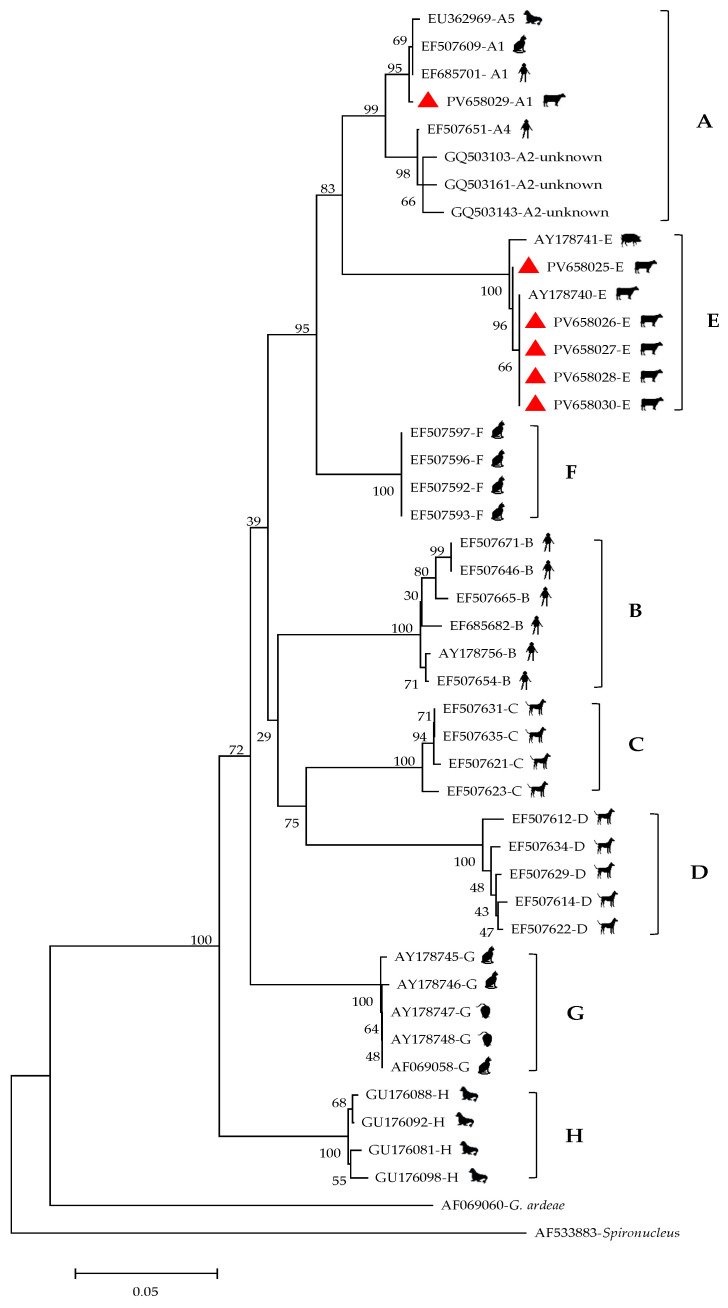
Phylogenetic tree of the *Giardia duodenalis* based on the *gdh* gene sequence. The red symbols are the samples in this study. Note: The reference sequences GU176088 and GU176092 are cited from reference [25].

Nested PCR analyses of the *gdh* loci revealed that 19 of the 529 fecal samples (3.6% (95% CI: 2.00–5.18%; 19/529)) were positive for *G. duodenalis*, with infection rates ranging from 0.9% to 7.5% among the four sampling locations (Table 4). The infection rate in Kunming was the highest (7.5% (95% CI: 3.37–11.63%; 12/160)), followed by Lincang (5.9% (95% CI: 1.25–10.63%; 6/101)) and Xishuangbanna (0.9% (95% CI: −0.93–2.81%; 1/106)). No *G. duodenalis* was detected in the 162 samples collected from Dehong. Among the seven beef cattle breeds, the detection rate ranged from 1.0% to 6.3%, with Aberdeen Angus cattle showing the highest rate (6.3% (95% CI: 0.83–11.67%; 5/80)), followed by Simmental (5.6% (95% CI: 2.48–8.63%; 12/216)), Hereford (4.8% (95% CI: −5.17–14.70%; 1/21)), and Brahman (0.9% (95% CI: −0.93–2.81%; 1/106)). The prevalence of *G. duodenalis* varied significantly across four age groups (1.1–24.1%), with the highest rate observed in pre-weaned calves (24.1% (95% CI: 7.57–40.70%; 7/29)), followed by weaned calves (6.5% (95% CI: 0.16–12.74%; 4/62)), young cattle (5.4% (95% CI: 0.13–10.68%; 4/74)), and adult cattle (1.1% (95% CI: 0.02–2.17%; 4/364)). In terms of gender, the infection rate was higher in females (4.4% (95% CI: −1.86–7.02%; 11/248)) than in males (2.8% (95% CI: 0.89–4.80%; 8/281)). Statistical analysis revealed significant differences among different regions and age groups (*p* < 0.001), while no significant variations were observed for the other three factors (breed, or sex). Of the positive samples in this study, 18 were identified as assemblage E, while only 1 sample (from an Aberdeen Angus cattle in Lincang) belonged to assemblage A. Phylogenetic analysis showed that among the obtained assemblage, 18 clustered within assemblage E, while a single sample was classified as assemblage 1 (Figure 3). The representative sequences have been submitted to GenBank and assigned GenBank accession numbers PV658025 to PV658030.

## 4. Discussion

This study provides new molecular epidemiological insights into the occurrence and genotype distribution of *E. bieneusi* and *G. duodenalis* in beef cattle in Yunnan Province, China. By combining nested PCR and sequence-based genotyping, we identified zoonotic genotypes of both parasites, albeit at relatively low infection rates. These findings contribute to the limited regional data available on parasitic protozoa in southwestern China and offer a comparative basis for understanding their epidemiological features across geographic regions. The implications of these results are multifaceted, spanning public health, veterinary parasitology, and livestock production.

The overall infection rate of *E. bieneusi* in Yunnan beef cattle was 3.0% (16/529), which is lower than the global pooled prevalence reported in cattle (12.9–16.6%) [26,27,28]. Furthermore, the prevalence observed in this study was lower than that reported in cattle from Asia (14.2%, 2000/14,132), Europe (16.2%, 71/441), the Americas (19.1%, 707/3703), Africa (13.2%, 20/152), and Oceania (10.4%, 49/471) [26]. Compared with other countries, the infection rate observed in this study were lower than the infection rates reported in most nations, including South Africa (7.4%), Egypt (6.1%), Thailand (5.0%), the U.S. (12.9%), Australia (10.4%), Türkiye (10.3%), South Korea (15.6%), Iran (18.7%), and Brazil (17.4%) [26]. Additionally, it was lower than the pooled prevalence of *E. bieneusi* in Chinese cattle (11.2–20.0%) [26,28,29,30] and lower than rates reported in Ningxia (45.9%, 50/109) [31], Jilin (37.6%, 35/93) [32], Hunan (32.3%, 144/446) [33], Heilongjiang (29.0%, 93/321) [34], Shanghai (26.5%, 214/809) [35], Henan (24.4%, 214/879) [31], Gansu (22.6%, 320/1414) [36], Shanxi (22.4%, 90/401) [37], Shaanxi (19.7%, 73/371) [38], Tianjin and Hebei (19.4%, 202/1040) [39], Xinjiang (16.5%, 85/514) [40], Zhejiang (14.0%, 37/265) [41], Jiangsu (13.0%, 177/1366) [42], Guangdong (11.1%, 160/1440) [36], Hainan (9.9%, 31/314) [43], Qinghai (7.2%, 40/554) [44], Jiangxi (5.4%, 30/556) [45], Anhui (4.2%, 40/955) [46], and Shandong (3.1%, 21/673) [35]. However, it was higher than rates reported in Yunnan (0.6%, 5/841) [20], Gansu (1.1%, 4/353) [47], Heilongjiang (1.4%, 22/1632) [48], and Tibet (2.5%, 11/442) [49].

The overall infection rate of *G. duodenalis* in cattle in Yunnan Province was 3.6% (19/529). Compared with other countries, this study’s overall infection rate was lower than that reported in the United States (52.0%, 237/456) [50], Scotland (32.5%, 126/388) [51], Maryland, USA (32.1%, 125/390) [52], India (12.5%, 9/72) [53], and Malaysia (8.3%, 10/120) [54], but higher than that in New Zealand (2.0%, 2/100) [55]. In China, the detection rate of *G. duodenalis* varies between provinces, ranging from 1.0% to 41.2% [56]. The detection rate in this study was similar to that in Tibet (3.8%, 17/442) [57] and lower than in Sichuan (41.2%, 126/306) [58], Inner Mongolia (29.5%, 149/505) [57], Yunnan (27.5%, 144/524) [21], Inner Mongolia (9.2%, 10/108) [59], Hubei (22.6%, 70/309) [60], Jiangsu (20.6%, 281/1366) [53], Shaanxi (16.8%, 29/173) [52], Xinjiang (13.4%, 69/514) [61], Qinghai (10.0%, 39/389) [62], and Henan (7.2%, 128/1777) [63], but higher than in Liaoning (3.1%, 3/98) [64], Guangdong (2.2%, 31/1440) [65], Ningxia (2.1%, 29/1366) [66], and Gansu (1.0%, 14/1414) [67].

The relatively low prevalence of *E. bieneusi* and *G. duodenalis* observed in this study may be partially attributed to the regional characteristics of cattle farming in Yunnan Province. In contrast to intensively managed, large-scale feedlots common in other parts of China or abroad, beef cattle production in Yunnan remains predominantly smallholder-based, with limited degrees of intensification and low animal stocking densities. Extensive management practices and free-range systems reduce close contact among animals, thereby potentially limiting the fecal–oral transmission of enteric protozoa such as *E. bieneusi* and *G. duodenalis* [49]. Moreover, the reduced use of shared water sources and confinement structures in these low-density farms may decrease environmental contamination and cyst/spore accumulation, further contributing to the low detection rates. These findings underscore the influence of production systems and farm ecology on pathogen circulation dynamics in livestock populations.

The four *E. bieneusi* genotypes (I, J, BEB4, BEB8) belonging to Group 2, which are associated with confirmed zoonotic transmission, have been identified in this study [12,15]. These genotypes were originally considered as host-specific to ruminants [3], but now demonstrate an expanding host range [68]. For instance, genotype I has been detected in diarrheic children and non-human primates in China [32,69,70]. Genotype J exhibits broad distribution in children, non-human primates, commercial broiler chickens, wild deer and pigeons [3,71,72,73,74,75,76]. Genotypes BEB4 and BEB8 have been identified in human infections, non-human primates and swine populations [3]. Notably, the four genotypes detected in our study are zoonotic genotypes identified in both humans and animals, posing potential public health risks [25,77]. In the current study, genotype I was the predominant genotype among the four detected, which is consistent with findings from Shanxi [37], Shaanxi [38], Tibet [49], and Zhejiang [78]. Genotype J is the most frequently reported genotype in most Chinese provinces [79], while genotype BEB4 has a relatively higher distribution in Qinghai [80]. Thus, our study suggests the zoonotic potential of all genotypes of *E. bieneusi* in Yunnan beef cattle farms.

Assemblages E and A of *G. duodenalis* were detected in this study. Consistent with previous findings, assemblage E is the predominant genotype of *G. duodenalis* infections in cattle, a finding consistent with most domestic studies, including those from Heilongjiang [56], Henan [63], Jiangxi [81], and Ningxia [82]. Although assemblage E has traditionally been considered as infecting animals, recent studies suggest that domestic cattle may serve as a source of *G. duodenalis* transmission to humans. For instance, in a rural Egyptian community with intensive cattle farming, 62.5% (25/40) of children tested positive for assemblage E, likely due to direct contact with livestock or exposure to contaminated water [83]. Additionally, reports of assemblage E infections in humans have shown a rising trend globally [84,85]. The assemblage A identified in this study belonged to the A1 subtype, which has been primarily detected in animals [86,87]. At the *gdh* locus, subtypes A1 and A5 primarily infect animals, while subtypes A2, A3, and A4 are predominantly associated with human infections. Subtype A6 has been predominantly identified in wild ruminants (primarily deer) [88]. However, human infections with the A1 subtype have also been reported in China [89], Portugal [90], Mexico [91], and Brazil [92]. Although only assemblage E and a minor proportion of assemblage A were detected in this study, *G. duodenalis* still poses a zoonotic risk in Yunnan beef cattle farms.

From a One Health perspective, these findings have both public health and economic implications. To meet its increasing meat consumption needs, Yunnan has aggressively expanded its beef cattle industry. However, it is important for both farmers and consumers to prioritize the prevention of bovine microsporidiosis and giardiasis, as these pathogens can enter the environment through feces, contaminating water sources, food, and soil. Human exposure to infectious spores/cysts can lead to infection, with immunocompromised individuals facing higher risks and more severe symptoms. Currently, there are no effective treatments for these two parasitic diseases. Factors such as climate, sanitation conditions, stocking density, and animal immunity influence the transmission of *E. bieneusi* and *G. duodenalis* in cattle. To effectively control these infections, it is essential to adopt the One Health approach, which integrates human, animal, and environmental interactions, understanding how these interactions occur and the factors sustaining infections in animals [93,94,95]. In the age of misinformation, it is crucial to promote public understanding of zoonotic parasites through accessible platforms, such as Twitter, TikTok and ResearchGate.

## 5. Conclusions

In conclusion, this study expands the current understanding of *E. bieneusi* and *G. duodenalis* infection dynamics in beef cattle in Yunnan Province, identifying genotypes with zoonotic potential. While the infection rate was relatively low, the presence of genotypes shared with humans warrants closer monitoring. Four genotypes of *E. bieneusi* (I, J, BEB8, BEB4) were identified, of which I, J, and BEB4 have been reported in both human and animal infections, highlighting their potential zoonotic relevance. In particular, genotype I was the most frequently detected in this study. For *G. duodenalis*, the majority of detected assemblages belonged to assemblage E, and one assemblage A, which has potential zoonotic transmission risks, was also identified. Future research should adopt longitudinal and multisite approaches, integrate environmental and host-level risk factors, and explore the role of antimicrobial resistance. At the same time, researchers should engage broader audiences through digital platforms to strengthen awareness of zoonotic parasites, align with One Health objectives, and enhance the societal relevance of veterinary parasitology.

## Figures and Tables

**Table 1:** Geographical distribution and sample collection details of seven species of beef cattle (Total *n* = 529) at four locations in Yunnan Province, China, for *Enterocytozoon bieneusi* and *Giardia duodenalis* investigations.

Location	Geographical Coordinates	Altitude (m)	No. of Samples							Total
			Simmental Cattle	Brahman Cattle	Aberdeen Angus Cattle	Yunnan Yellow Cattle	Humped Cattle	Dulong Cattle	Hereford Cattle	
Dehong	98°48′ E, 24°33′ N	835–2800	87	-	-	39	36	-	-	162
Kunming	102°91′ E, 25°63′ N	2100–2900	129	-	-	-	-	31	-	160
Xishuangbanna	101°55′ E, 21°52′ N	640–1200	-	106	-	-	-	-	-	106
Lincang	99°95′ E, 24°14′ N	740–1400	-	-	80	-	-	-	21	101
Total	-	-	216	106	80	39	36	31	21	529

Note: Dash (-) indicates no samples collected for that breed at the location. Altitude ranges show minimum–maximum elevations of sampling sites.

**Table 2:** Information on the primer used for the molecular identification and/or the characterization of *Enterocytozoon bieneusi* and *Giardia duodenalis* in the present study.

Species	Loci	Primer ID	Primer Sequences (5′-3′)	Fragment Length (bp)	Temperature Annealing (°C)	Reference
*Enterocytozoon* *bieneusi*	ITS gene	EB-F1	GGTCATAGGGATGAAGAG	392	55 °C	[23]
		EB-R1	TTCGAGTTCTTTCGCGCTC			
		EB-F2	GCTCTGAATATCTATGGCT			
		EB-R2	ATCGCCGACGGATCCAAGTG			
*Giardia* *duodenalis*	*gdh* gene	gdh-F1	TTCCGTRTYCAGTACAACTC	530	59 °C	[24]
		gdh-R1	ACCTCGTTCTGRGTGGCGCA			
		gdh-F2	ATGACYGAGCTYCAGAGGCACGT			
		gdh-R2	GTGGCGCARGGCATGATGCA			

**Table 3:** Occurrence, factors and genotypes associated with *Enterocytozoon bieneusi* infection in beef cattle in Yunnan Province, China.

**Variable**	**Category**	**No.** **Tested**	**No.** **Positive**	**Prevalence (%)** **(95% CI)**	**OR (95%, CI)**	***p*-Value**	**Genotype**
Region	Kunming	160	7	4.4 (1.17–7.58)	1.49 (0.38–5.92)	0.207	I (4), BEB8 (3)
	Dehong	162	6	3.7 (0.76–6.64)	1.26 (0.31–5.14)		J (3), I (2), BEB4 (1)
	Xishuangbanna	106	-	-	-		-
	Lincang	101	3	3.0 (−0.40–6.34)	Reference		I (2), J (1)
Breed	Simmental cattle	216	12	5.6 (2.48–8.63)	2.06 (0.26–16.34)	0.088	I (6), J (3), BEB8 (3)
	Brahman cattle	106	-	-	-		-
	Aberdeen Angus	80	3	3.8 (−0.50–8.00)	1.36 (0.14–13.58)		I (2), J (1)
	Yunnan Yellow cattle	39	-	-	-		-
	Humped cattle	36	1	2.8 (−2.86–8.42)	Reference		BEB4 (1)
	Dulong cattle	31	-	-	-		-
	Hereford cattle	21	-	-	-		-
Gender	Female	248	10	4.0 (1.57–6.50)	1.93 (0.69–5.38)	0.204	I (5), J (2), BEB8 (3)
	Male	281	6	2.1 (0.43–3.84)	Reference		I (3),J (2), BEB4 (1)
Age	Pre-weaned (0–60 days)	29	6	20.7 (5.01–36.37)	31.39 (7.37–133.64)	<0.001	I (4), BEB8 (2)
	Post-weaned (61–180 days)	62	2	3.2 (−1.30–7.75)	4.01 (0.66–24.51)		J (1), BEB8 (1)
	Juvenile cattle (7–18 months)	74	5	6.8 (0.90–12.61)	8.72 (2.04–37.34)		J (3), I (2)
	Adult cattle (>18 months)	364	3	0.8 (−0.11–1.76)	Reference		I (2), BEB4 (1)
Total		529	16	3.0 (1.56–4.49)	-	-	I (8), J (4), BEB8 (3), BEB4 (1)

No: number; CI: confidence interval; OR: odds ratio.

**Table 4:** Occurrence, factors and assemblages associated with *Giardia duodenalis* infection in beef cattle in Yunnan Province, China.

Variable	Category	No. Tested	No. Positive	Prevalence (%) (95% CI)	OR (95%, CI)	*p*-Value	Assemblages
Region	Kunming	160	12	7.5 (3.37–11.63)	8.51 (1.09–66.48)	<0.001	E (12)
	Dehong	162	-	-	-		-
	Xishuangbanna	106	1	0.9 (−0.93–2.81)	Reference		E (1)
	Lincang	101	6	5.9 (1.25–10.63)	6.63 (0.78–56.09)		E (5), A (1)
Breed	Simmental cattle	216	12	5.6 (2.48–8.63)	6.18 (0.79–48.15)	0.116	E (12)
	Brahman cattle	106	1	0.9 (−0.93–2.81)	Reference		E (1)
	Aberdeen Angus	80	5	6.3 (0.83–11.67)	7.00 (0.80–61.15)		E (4), A (1)
	Yunnan Yellow cattle	39	-	-	-		-
	humped cattle	36	-	-	-		-
	Dulong cattle	31	-	-	-		-
	Hereford cattle	21	1	4.8 (−5.17–14.70)	5.25 (0.32–87.44)		E (1)
Gender	Female	248	11	4.4 (1.86–7.02)	1.58 (0.63–4.00)	0.327	E (11)
	Male	281	8	2.8 (0.89–4.80)	Reference		E (7), A (1)
Age	Pre-weaned (0–60 days)	29	7	24.1 (7.57–40.70)	28.64 (7.79–105.25)	<0.001	E (7)
	Post-weaned (61–180 days)	62	4	6.5 (0.16–12.74)	6.21 (1.51–25.51)		E (3), A (1)
	Juvenile cattle (7–18 months)	74	4	5.4 (0.13–10.68)	5.14 (1.26–21.05)		E (4)
	Adult cattle (>18 months)	364	4	1.1 (0.02–2.17)	Reference		E (4)
Total		529	19	3.6 (2.00–5.18)	-	-	E (18), A (1)

No: number; CI: confidence interval; OR: odds ratio.

## Data Availability

The datasets presented in this study can be found in online repositories. The original contributions presented in this study are included in the article. Further inquiries can be directed to the corresponding authors.
